# Fructus Xanthii Attenuates Hepatic Steatosis in Rats Fed on High-Fat Diet

**DOI:** 10.1371/journal.pone.0061499

**Published:** 2013-04-09

**Authors:** Xiumin Li, Zhipeng Li, Mei Xue, Zhimin Ou, Ming Liu, Mingxing Yang, Suhuan Liu, Shuyu Yang, Xuejun Li

**Affiliations:** 1 Xiamen Diabetes Institute, The First Affiliated Hospital of Xiamen University, Xiamen, China; 2 Division of Endocrinology and Diabetes, The First Affiliated Hospital of Xiamen University, Xiamen, China; 3 Central Laboratory, The First Affiliated Hospital of Xiamen University, Xiamen, China; INRA, France

## Abstract

*Fructus Xanthii* (FX) has been widely used as a traditional herbal medicine for rhinitis, headache, cold, etc. Modern pharmacological studies revealed that FX possesses anti-inflammatory, anti-oxidative, and anti-hyperglycemic properties. The present study was designed to investigate the effects of FX on glucose and insulin tolerance, and hepatic lipid metabolism in rats fed on high-fat diet (HFD). Hepatic steatosis was induced by HFD feeding. Aqueous extraction fractions of FX or vehicle were orally administered by gavage for 6 weeks. Body weight and blood glucose were monitored. Glucose and insulin tolerance test were performed. Liver morphology was visualized by hematoxylin and eosin, and oil red O staining. Expression of liver lipogenic and lipolytic genes was measured by real-time PCR. We showed here that FX improved glucose tolerance and insulin sensitivity in HFD rats. FX significantly decreased the expression of lipogenic genes and increased the expression of lipolytic genes, ameliorated lipid accumulation and decreased the total liver triglyceride (TG) content, and thus attenuated HFD-induced hepatic steatosis. In conclusion, FX improves glucose tolerance and insulin sensitivity, decreases lipogenesis and increases lipid oxidation in the liver of HFD rats, implying a potential application in the treatment of non-alcoholic fatty liver disease.

## Introduction

The worldwide incidence of obesity has increased dramatically during recent decades. The “thrifty genes”, which were historically advantageous, may now be detrimental due to the abundance of the food supply and dramatic changes in modern lifestyle, and contribute to the major epidemic of obesity [1]. Obesity is associated with a high incidence of steatosis, insulin resistance and chronic inflammation [2]. Obesity-related non-alcoholic fatty liver disease (NAFLD) has recently been recognized as one of the major causes of chronic liver disorders [3], estimated to affect at least one-quarter of the general population [4]. NAFLD is characterized by excess liver lipid accumulation, hepatic insulin resistance, and later hepatic inflammation, leading to nonalcoholic steatohepatitis and culminating in hepatic fibrosis or cirrhosis [5].

One of the major causes of fat accumulation in NAFLD is the inability of the liver to regulate the changes in lipogenesis in the transition from fasted to fed state [6]. Several studies suggested that hepatic lipogenesis is increased in hepatic steatosis, which may result from either increased triglyceride synthesis or decreased fatty acid oxidation through production of malonyl- CoA, both leading to increased triglyceride content in the liver [7]. Excess fat accumulation ultimately leads to hepatic steatosis and worsening hepatic insulin resistance via a network of transcription factors [8], which regulate hepatic lipogenesis and fatty acid oxidation, including carbohydrate responsive element binding protein (ChREBP), sterol regulatory element-binding protein-1c (SREBP-1c), liver X receptor (LXR), and peroxisome proliferator-activated receptor (PPAR).

ChREBP, controlling 50% of hepatic lipogenesis by regulating glycolytic and lipogenic gene expression [9], plays important roles in the regulation of de novo lipogenesis and in the development of fatty liver [10]. SREBP is a transcription factor that controls genes involved in cholesterol uptake and biosynthesis. In addition, the SREBP-1c isoform has been shown to regulate the transcription of genes of lipogenic pathway [11]. Enzymes of the lipogenic pathway that are transcriptionally regulated include acetyl-CoA carboxylase (ACC), fatty acid synthase (FAS), and stearoyl-CoA desaturase (SCD-1).

LXRs, nuclear receptors controlling lipid metabolism, were discovered as sterol sensors, which regulate cholesterol homeostasis. In rodents, LXR promotes cellular cholesterol efflux, transport, and excretion [12]. However, LXR activation also induces hepatic steatosis. Further on, LXR was also shown to induce the expression of CD36, a fatty acid transporter, and scavenger receptor, suggesting another mechanism by which LXR can promote fatty liver [13]. Moreover, LXR can promote lipogenesis in an SREBP-1c independent manner and activate another lipogenic transcriptional factor ChREBP [14], [15].

LXR and PPAR were characterized as metabolism regulators. A direct interaction between LXRα and PPARα has been proposed [16], which would be a logical way of preventing the simultaneous activation of the opposing pathways [17]. LXR has been shown to strongly induce transcription of SREBP-1c gene via forming obligate heterodimers with retinoid X receptors (RXRs). It is thought that the transcription factor PPARα prevents the heterodimerisation of LXR with RXR [18].

PPARα target genes involved in β-oxidation include Carnitine-palmitoyltransferase-1 (CPT1), acyl-CoA oxidase (ACO), acyl-CoA oxidase 1 (ACOX1), and so on [19]. CPT1 regulates long-chain fatty acid entry into mitochondria for β-oxidation; ACO is the first step enzyme of the β-oxidation cycle; ACOX1 is the first and a rate-limiting enzyme of the PPARα regulated and peroxisome proliferator-inducible fatty acid β- oxidation system.

Chronic low-grade inflammation is considered to have a pivotal role in the development of obesity and associated metabolic disorders [20], [21]. The inflammatory response triggered by obesity involves many components of the classical inflammatory response and increases in inflammatory cytokines [22]. Pro-inflammatory cytokines, such as tumor necrosis factor (TNF)-α and interleukin (IL)-1β are implicated in the development of obesity-related systemic inflammation and NAFLD [23], [24].

*Fructus Xanthii* (FX), called Cang-Erzi in Chinese Pin Yin, was firstly recorded in Qian Jin dietetic therapy, and commonly used as a traditional Chinese medicine for treatment of sinusitis and rheumatic headache. Data from animal experiments validated that FX possesses antioxidant, anti-nociceptive, and anti-inflammatory properties, and can protect pancreatic beta-cells form cytokine-induced damage [25], [26]. However, there has been no investigation regarding the effects of FX on fat metabolism in high-fat diet (HFD)-induced NAFLD. The present study was designed to explore the effects of FX in treating HFD-induced hepatosteatosis, and its possible mechanisms.

## Materials and Methods

### Plant materials

The raw material of FX was provided by Department of Pharmacy, the First Affiliated Hospital of Xiamen University, China. The herbs were immersed in 10 times volume of water (V/V) for half an hour, and decocted twice at boiling temperature for 2 h. The decocted liquids were collected, filtrated, concentrated to 114 mg/ml, and were then placed in sterile bottle (30 ml) and stored in the refrigerator. Finger print analysis of FX was performed using High Performance Liquid Chromatography (HPLC) from 3 different batches of the FX preparation. The results showed same retention time and similar peak area (Fig. S1 and Table S2), manifesting that the composition of the FX decoction is consistent and reproducible.

### Experimental animals

Male Sprague-Dawley rats, weighing 180–200 g, were purchased from Shanghai Experimental Animal Center, Chinese Academy of Sciences (Shanghai, China). The rats were housed in temperature and humidity-controlled room, kept on 12 h light/dark cycle and provided with unrestricted amount of rodent chow and water. After acclimatization for 1 week, the rats were randomly allocated into four groups: normal control diet (NCD), high fat diet (HFD: 20% lard, 4% sucrose, 2% milk, 1% cholesterol, and 73% standard chow), HFD treated with low (570 mg/kg/d) and high-dose (1140 mg/kg/d) of FX respectively. FX was given orally between 9:00–10:00 am once daily, from 6 weeks of HFD consumption till 12 weeks. The rats in NCD and HFD groups received equivalent volume of vehicle. After 6 weeks of FX treatment, the rats were anesthetized with pentobarbital (40 mg/kg body weight). The blood was collected via cardiac puncture; tissues were removed, weighted, and stored at −80 °C. After tissue harvest, the rats were sacrificed under deep anesthesia. To assess the liver toxicity of FX in normal chow diet rat, a group of mice was treated with FX at high dose (1140 mg/kg/d) for 14 days. Serum levels of alanine transaminase (ALT) and aspartate transaminase (AST), and gene expression of lipid metabolism regulators were evaluated. All animal experiments were approved by Xiamen University Animal Care and Use Committee.

### Oral glucose tolerance test (OGTT)

After an overnight fasting, OGTT was performed using OneTouch Ultra Blood Glucose Meter (LifeScan, USA). Glucose was administrated by gavage (2 g/kg body weight) and blood glucose was measured at time points of 0, 30, 60, 90 and 120 min, post gavage. Glucose areas under the curve (AUC) values during the OGTT were calculated using the trapezoidal rule [27].

### Intraperitoneal insulin tolerance test (IPITT)

After a 6 h fasting, the rats were given an intraperitoneal injection of insulin (0.5 U/kg body weight). Blood glucose was measured before and at 15, 30, 45, 60, 90, and 120 min after insulin injection. Glucose areas under the curve (AUC) values during the IPITT were calculated using the trapezoidal rule [27].

### Histological staining and oil red O staining

Abnormal lipid metabolism in the liver leads to triglyceride (TG) accumulation and fatty liver formation. The liver morphology was visualized by hemotoxylin and eosin (H&E) staining. The sections of liver were fixed in 10% formalin, dehydrated, embedded in paraffin and stained with H&E. For oil red O staining, a stock solution of oil red O (0.5 g/100 ml) in isopropanol was prepared, stored and protected from light. Liver tissue was embedded in Optimal Cutting Temperature gel. Air-dried tissue sections of 7 µm were dipped in formalin, washed with oil red O without counterstaining with hematoxylin.

### Measurement of serum ALT, AST and liver TG content

The levels of ALT and AST were measured by automatic biochemistry analyser (Architect c8000, USA) following the manufacturer's instructions.

Excessive accumulation of TG in the liver is the hallmark of NAFLD. For hepatic TG level, 100 mg of liver tissue was homogenized for extraction of lipid by Triglyceride Quantification Kit (Biovision, USA). Total lipid was resuspended in 5% NP-40 in water, and level of TG was then quantified according to the manufacturer's procedures. The protein concentration was determined using BCA protein assay kit (Thermo Scientific, USA). The data was expressed as nmol/ng protein.

### Measurement of serum insulin

Serum insulin was measured using an enzyme-linked immunosorbent assay (ELISA) kit (Millipore, USA) according to the manufacturer's instructions.

### RNA extraction and RT-PCR

Total RNA was extracted using RNAsimple Total RNA Kit (Tiangen Biotech, China). Reverse transcription of total RNA to cDNA was carried out with PrimeScript RT reagent kit with gDNA eraser (TaKaRa, China) in a MyCycler Thermal Cycler (Bio-Rad, USA) following the manufacturer's instructions.

Real-time quantitative PCR was performed with LightCycler 480 SYBR Green I Master Mix (Roche Diagnostics GmbH, Germany) in a Light Cycler 480 System. PCR reaction was performed following the cycling protocol of 95°C for 5 min, followed by 45 PCR cycles with 95 °C for 5 s, 58 °C for 15 s and 72 °C for 20 s. Dissociation curves were run after amplification to identify the specific PCR products. Light Cycler 480 Software was employed to perform the relative quantification for the expression of target genes. The relative mRNA transcript levels were calculated according to the 2^−ΔΔCp^ (Cp  =  crossing point) method. The primers used were shown in Table S1.

### Statistical analysis

All values are expressed as mean ± SE. All statistical analysis was performed using one-way analysis of variance (ANOVA) or Student's *t*-test when applicable. P<0.05 was accepted as statistically significant.

## Results

### FX decreased liver weight

**Figure 1:**
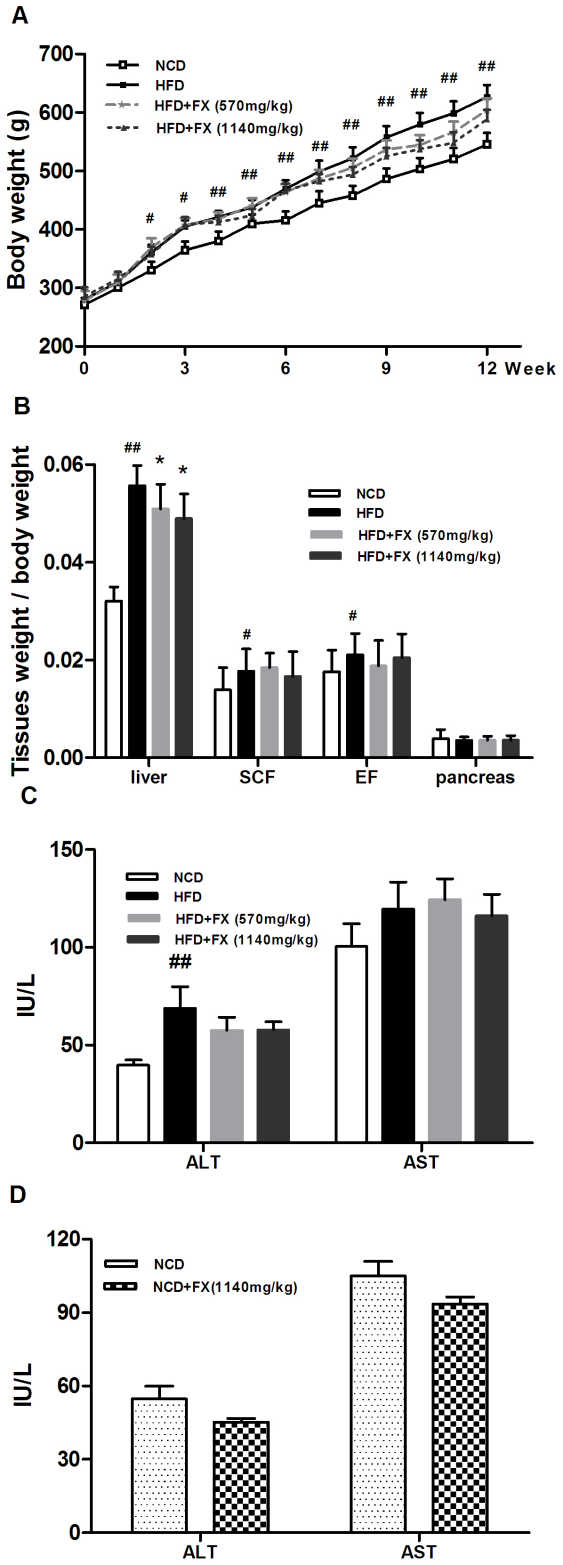
Effects of FX on body weight, relative tissue weight and serum ALT and AST levels in rats. Body weight (A), relative tissue weight (B), and serum levels of ALT and AST of rats fed on HFD (C) and on NCD (D). Data given are mean ± SE. (N = 10, ^#^P<0.05, ^##^P<0.01 vs. NCD group; *P<0.05 vs. HFD group. SCF: subcutaneous fat; EF: epididymal fat).

FX tended to decrease body weight after 7-week treatment, although the trend was not statistically significant (Fig. 1). The weight of various tissues, including subcutaneous fat (SCF), epididymal fat (EF), and pancreas, were similar among groups. The liver weight of FX groups was significantly lower (P<0.05) than that of HFD group, showed as relative liver weight (Fig. 1B). FX has been suggested to have hepatotoxicity at high doses. Here, we showed that HFD alone increased the serum levels of ALT (P<0.01). However, FX treatment at low and high dose did not have further detrimental effects, and this was also true in normal chow diet rats (Fig. 1C and D). In addition, FX even tends to lower the ALT level in rats fed on normal chow diet (P = 0.05) (Fig. 1D), implying a liver protective effect.

### FX improved glucose tolerance and insulin sensitivity

**Figure 2:**
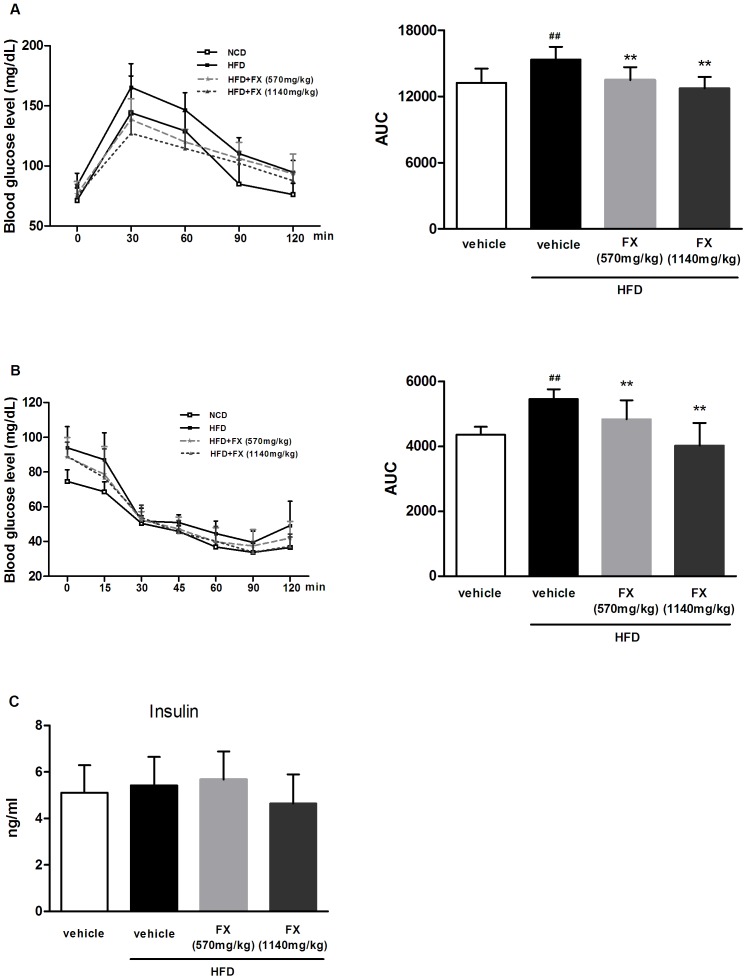
Effects of FX on glucose and insulin tolerance. Glucose level in OGTT (A), in IPITT (B), and serum insulin level (C). AUC: blood glucose area under the curve. Data given are mean ± SE, N = 10. ^##^P<0.01 vs. NCD group; **P<0.01 vs. HFD group.

After 6-week of HFD feeding, blood glucose increased in the OGTT and IPITT test, revealing impaired glucose tolerance and insulin sensitivity (data not shown). After administration of FX for 6 weeks, blood glucose levels were significantly lower at 30 and 60 min post glucose-load in HFD rats treated with FX compared to that in HFD group, and the AUC of FX (both high and low doses) groups was decreased (P<0.01) (Fig. 2A). IPITT was performed after 6 weeks of FX administration, and the results revealed that blood glucose markedly decreased in FX treated rats compared to vehicle (P<0.01) (Fig. 2B), suggesting improved insulin sensitivity. The serum level of insulin was not different among groups (Fig. 2C).

### FX inhibited lipid accumulation in liver

**Figure 3:**
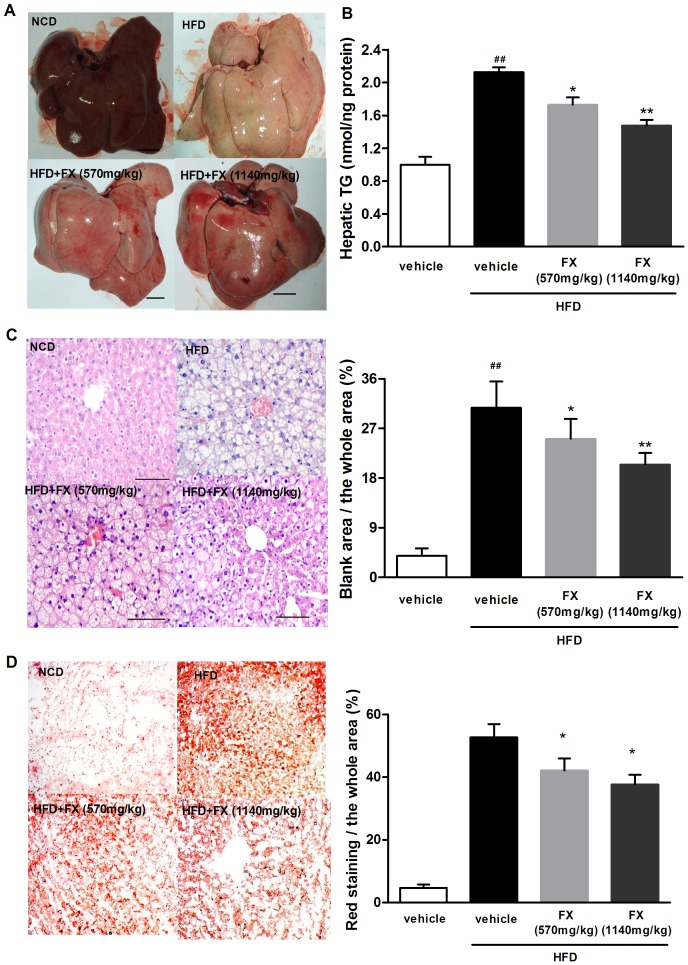
Effects of FX on lipid accumulation in the liver. Macroscopic images of liver (A), TG content in the liver (B), liver sections stained with H&E (C) and oil red (D). Data given are mean ± SE. N = 10, ^##^P<0.01 vs. NCD group; *P<0.05, **P<0.01 vs. HFD group. The size bar corresponds to 1.5 cm in (B) and 100 µm in (C). Zoom: 40×in (D).

Liver TG content in the HFD group was increased by 2 fold compared to the NCD group (P<0.01). FX treated rats showed decreased hepatic TG content compared to that of HFD rats (P<0.05) (Fig. 3B). Gross morphological differences of liver were shown in Fig. 3A, where lipid accumulation in liver resulted in pale discoloration. As shown in Fig. 3C, HFD consumption increased liver lipid content, which was significantly attenuated by FX treatment. This finding was further confirmed by assessment of lipid accumulation using oil red O staining (Fig. 3D).

### FX improved lipid metabolism

**Figure 4:**
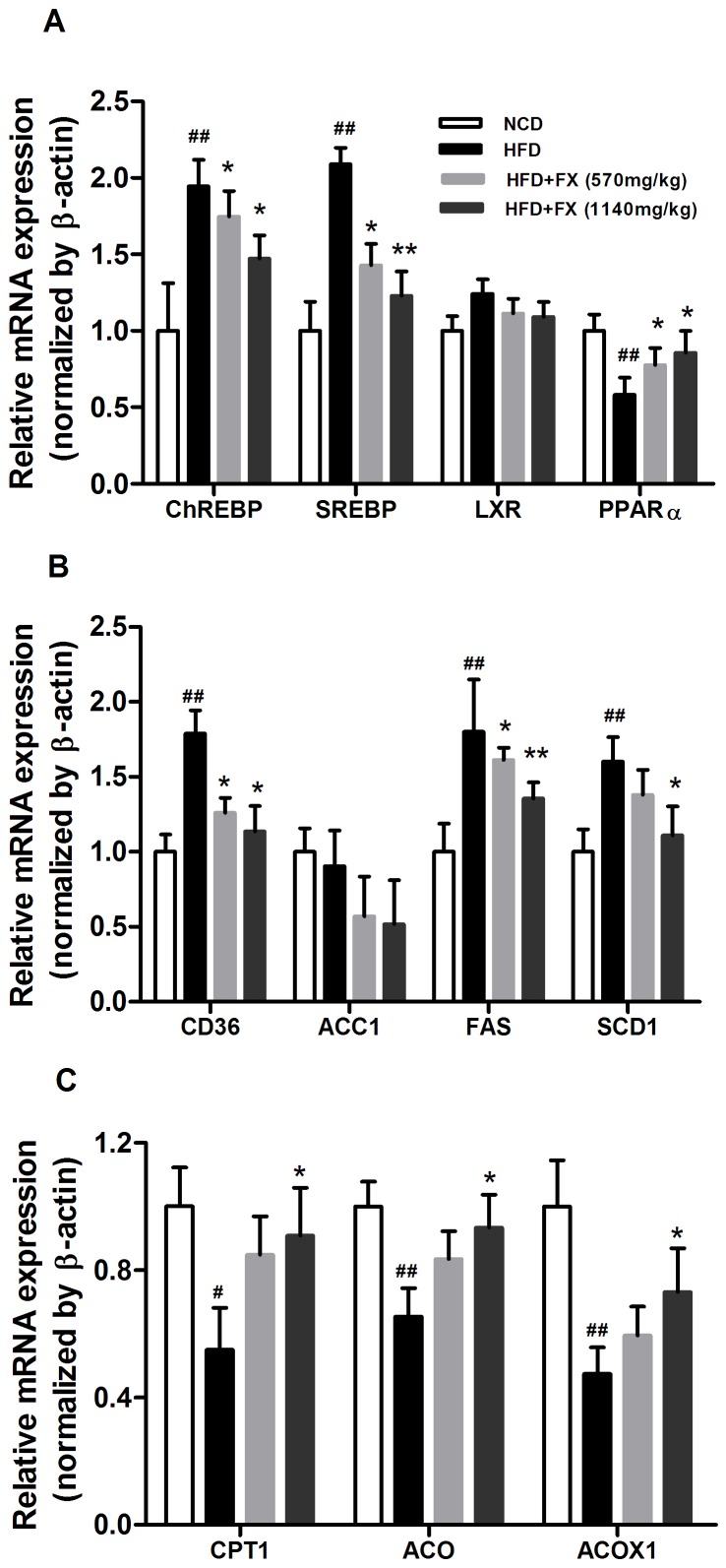
Gene expression of lipid metabolism regulators. Relative expression of transcription factors (A), lipid transport and lipogenic genes (B), fatty acid oxidation genes (C) relative to β-actin in liver, and normalized by NCD group. Data given are mean ± SE. N = 10, ^#^P<0.05, ^##^P<0.01 vs. NCD group; *P<0.05, **P<0.01 vs. HFD group.

Liver expression of ChREBP and SREBP-1c expression was significantly increased by around 2 fold in HFD rats compared to NCD rats, respectively (P<0.01), and was attenuated by FX treatment in a dose-dependent manner (P<0.05). HFD decreased mRNA level of PPARα (P<0.01), which was reversed by FX treatment (P<0.05) (Fig. 4A). HFD-induced ectopic lipid accumulation in the liver challenged the normal lipid transport and metabolism (Fig. 4 B). CD36 mRNA level was increased in HFD rats compared to that of NCD rats (P<0.01), which was suppressed by FX treatment (P<0.05). The mRNA levels of FAS and SCD1 were significantly lower in FX treated rats compared to that of HFD rats. FX dose dependently decreased FAS expression (P<0.05). High dose FX treatment lowered SCD1 expression (P<0.05). There was no difference in ACC1 expression between NCD and HFD rats, while the levels of ACC1 of FX groups tend to be lower than that of HFD group, though not reaching statistical significance (P>0.05). As shown in Fig. 4C, the gene expression of CPT1, ACO and ACOX1 was decreased in HFD rats compared with that of NCD rats (P<0.05), which were reversed by FX treatment (P<0.05). Regulation of lipid metabolism genes by FX was not observed in normal chow diet rats (Supplemental Fig. S2).

### FX decreased the gene expression of inflammatory cytokines in the liver

**Figure 5:**
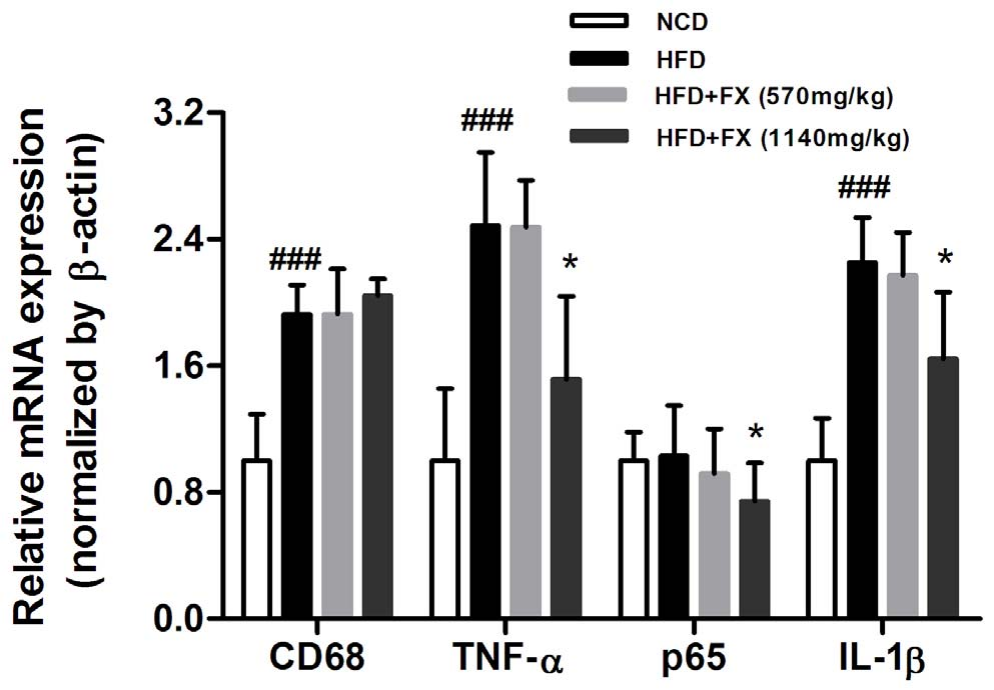
Relative gene expression of inflammatory cytokines in the liver. Gene expression was normalized by NCD group. Data given are mean ± SE. N = 10, ^###^P<0.001 vs. NCD group; *P<0.05 vs. HFD group.

The gene expression of CD68 was increased in HFD-fed rats, indicating an increased macrophage infiltration. In HFD rats, TNF-α and IL-1β were significantly increased around 2 fold as compared to that of NCD (P<0.001). FX treatment at high dose significantly reversed HFD-induced increase of TNF-α and IL-1β gene expression, suggesting an anti-inflammatory effect (Fig. 5), which was absent in low dose FX treated rats.

## Discussion

Traditional Chinese Medicines has been playing important roles in ancient and modern Chinese medical practice. FX, a commonly used herb medicine in traditional Chinese medicine for treating multiple aliments, has been shown to possess antioxidant, anti-nociceptive, and anti-inflammatory properties [25], [26]. Here we showed that FX decreased fatty acid synthesis and lipid accumulation in the liver, thus attenuated hepatic steatosis in HFD rats, through inhibiting lipogenesis and promoting lipolysis mechanisms.

Decrease of hepatic lipid accumulation is a rational target for NAFLD therapy since lipid loss should reduce many of the putative mediators of liver injury including insulin resistance, hepatic free fatty acid supply and pro-inflammation [28]. The nature of FX anti-inflammatory and control of blood glucose could be by minimizing the low-grade or chronic inflammation in NAFLD [21] and insulin resistance resulting from lipid accumulation in liver [9].

The liver is an important principal organ in the maintenance of glucose homeostasis and energy storage for the conversion of excess dietary nutrients into triglycerides. Under HFD feeding conditions, excess TG in the liver induces fatty liver and eventually insulin resistance. HFD feeding increased the body weight gradually along the time course, 2 weeks after HFD. HFD rats body weight was significantly higher compared to NCD rats, and FX treatment tended to decrease the body weight although not significantly. The weight of various tissues, including SCF, EF, and pancreas, was similar amongst all groups. The liver weight and relative liver weight in FX treated rats were significantly lower than that of HFD rats, suggesting that FX has a beneficial effect on lowering TG content in the liver. It has been shown that FX has hepatotoxicity at a high dosage, while the doses we used here didn't induce a further alteration of serum ALT and AST level compared to rats on HFD alone. When administrated to normal chow diet rats, FX even tended to decrease the ALT levels, implying a potential liver protective effect.

TG is thought to be a surrogate marker of disrupted insulin signal. In other words, hepatic insulin resistance is associated with the accumulation of TG and FA metabolites [8]. The rate of glucose disposal and insulin sensitivity were measured by OGTT and IPITT. FX treated rats had significantly lower levels of blood glucose after administration of an exogenous load of glucose, suggesting an enhanced glucose disposal. When challenged with excessive amounts of insulin, FX treatment showed drastically reduced level of blood glucose and improved glucose disposal in HFD rats, suggesting that FX increased insulin sensitivity. The raised insulin sensitivity could also reflect less lipid accumulation in the liver indirectly.

FX treatment significantly decreased liver TG content. Morphologically, the liver of HFD rats showed abundant and large lipid droplets, and obvious increase of liver derangement compared to that of NCD rats. However, the liver of FX rats had fewer lipid droplets and more normal liver morphology, suggesting a beneficial effect of FX on preventing lipid accumulation and reversal of disrupted structure of the liver. In addition, FX may also exert liver protective effect via inhibition of HFD-induced inflammation, since our results showed that FX decreased gene expression of inflammatory cytokines.

To explore the possible mechanisms of FX on decreasing liver lipids accumulation, we investigated the expression levels of several genes related to fatty acid transport, and lipid metabolism including lipogenesis and β-oxidation. ChREBP regulates the balance between glycogen and triglyceride storage by coordinately regulating glycolytic and lipogenic gene expression [9]. The results showed that the level of ChREBP was significantly higher in the liver of rats fed HFD compared with that of NCD. FX effectively inhibited the raise of ChREBP expression. Expression of ChREBP transcriptional targets FAS and ACC1 also strongly correlates with ChREBP expression. FAS catalyze the last step in fatty acid biosynthesis, and thus, it is believed to be a major determinant of the maximal hepatic capacity to generate fatty acids by de novo lipogenesis. FX markedly decreased the HFD-induced high expression of ACC1 and FAS. The trend of SCD1 influenced by FX was similar to that of FAS but only at high dose. There is evidence suggested that transgenic hepatic over-expression of SREBP-1c produced a fatty liver and a 4- fold increase in the rate of hepatic fatty acid synthesis with increase in lipogenic genes like FAS, ACC, and SCD [29]. Here, we showed that hepatic SREBP-1c mRNA in HFD rats was increased. FX treatment generated a significant decreasing effect on its expression paralleled with the change in ChREBP expression. LXR are unlikely to contribute to the down-regulation of ChREBP, SREBP-1c, and lipid metabolism related enzyme expression on HFD, because expression of LXR was only modestly increased. LXR is thought to induce CD36 expression. However, our results showed that hepatic CD36 mRNA level was significantly higher in HFD group compared with NCD group, and tended to be lower in FX groups than HFD group, albeit not significantly.

Our results indicated that hepatic CPT-1 mRNA expression was increased in the FX groups compared with HFD group. CPT-1 is the rate-limiting enzyme for fatty acid β-oxidation. If the CPT-1 mRNA was down-regulated in the hepatocyte, it might impair the energy substrate's transport, energy charge required for cell regeneration, and induce dyslipidemia with accumulation of fat in blood and hepatocytes. Therefore, we proposed that FX could promote the catabolism and utilization of fat through inducing an increased expression of CPT-1 mRNA. PPARs are proposed to play a central role in a signaling system that controls lipid homeostasis. FX regulated the function of PPAR, SREBP-1c, and ChREBP, decreased the mRNA levels of CD36, FAS, SCD-1, and increased the mRNA expression of CPT-1, ACO, and ACOX1, suggesting that FX plays an important and specific role in regulating fatty acid synthesis and oxidation by specifically controlling the expression of transcription factor SREBP-1c, ChREBP, PPARα and its down stream target genes.

In conclusion, we showed that FX has a beneficial effect in inhibiting fat accumulation in liver, improves insulin resistance and glucose disposal, inhibits inflammation, and possesses a repressive property on hepatic lipogenesis, which are associated with the inhibition of ChREBP and SREBP-1c, and induction of PPARα, suggesting a potential application of FX in treating fatty liver diseases.

## Supporting Information

Figure S1 **HPLC chromatograms of a standard solution (lower) and three batches of FX decoction (upper).** All chemicals were of analytical grades. Caffeic acid and chlorogenic acid were purchased from Sigma Chemicals (St. Louis, MO, USA). 3, 4- Dihydroxybenzoic acid, neochlorogenic acid, isochlorogenic acid C, cynarin and 4-dicaffenolyquinic acid were bought from Aldrich Chemical Company, Inc.HPLC separation was performed on C18 column (250 mm×4.6 mm. 5 µm). The mobile phase consisted of solvent A (methanol) and solvent B (water containing 0.2% formic acid). The gradient elution program was as follows: 0∼20 min, 2–5% A, 20∼30 min, 5–8% A, 30∼45 min, 8–15% A; 45∼55 min, 15–25%; 55∼60 min, 25%; 60∼70 min, 25–35% A; 70∼75 min, 35–38% A; 75∼95 min, 38–65% A; 95∼105 min, A was isocratic at 65%. The UV wavelength was set at 260 nm, column temperature was kept at 25 °C and the flow rate was set at 1.0 mL/min. Three batches of FX were detected at the same condition.
(TIF)

Figure S2 **Relative expression of lipogenic, lipolytic genes of liver in NCD treated with FX (1140 mg/kg).** Data given are mean ± SE. N = 5.
(TIF)

Table S1 **The oligonucleotide primers used.**
(TIF)

Table S2 **The retention time of the seven marker compounds in the three batches of FX decoction was shown as Mean ± SE.**
(TIF)
