# Supplementary figures and images for: Fructus Xanthii Attenuates Hepatic Steatosis in Rats Fed on High-Fat Diet
Source: PLoS One. 2013 Apr 9;8(4):e61499. doi: 10.1371/journal.pone.0061499 (PMC3621865; doi:10.1371/journal.pone.0061499)

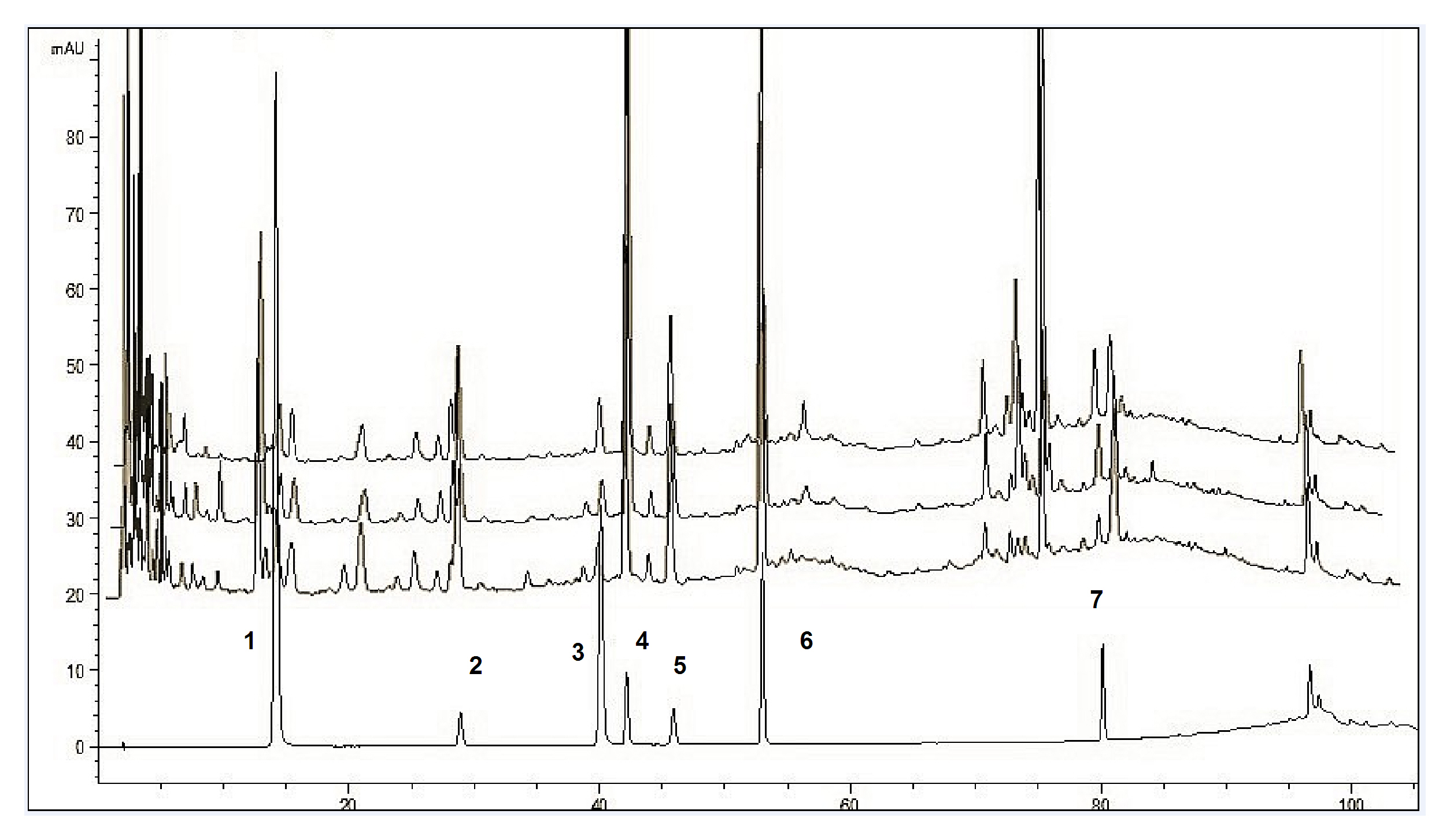

Supplement: Figure S1 — HPLC chromatograms of a standard solution (lower) and three batches of FX decoction (upper). All chemicals were of analytical grades. Caffeic acid and chlorogenic acid were purchased from Sigma Chemicals (St. Louis, MO, USA). 3, 4- Dihydroxybenzoic acid, neochlorogenic acid, isochlorogenic acid C, cynarin and 4-dicaffenolyquinic acid were bought from Aldrich Chemical Company, Inc.HPLC separation was performed on C18 column (250 mm×4.6 mm. 5 µm). The mobile phase consisted of solvent A (methanol) and solvent B (water containing 0.2% formic acid). The gradient elution program was as follows: 0∼20 min, 2–5% A, 20∼30 min, 5–8% A, 30∼45 min, 8–15% A; 45∼55 min, 15–25%; 55∼60 min, 25%; 60∼70 min, 25–35% A; 70∼75 min, 35–38% A; 75∼95 min, 38–65% A; 95∼105 min, A was isocratic at 65%. The UV wavelength was set at 260 nm, column temperature was kept at 25 °C and the flow rate was set at 1.0 mL/min. Three batches of FX were detected at the same condition. (TIF) [file pone.0061499.s001.tif]

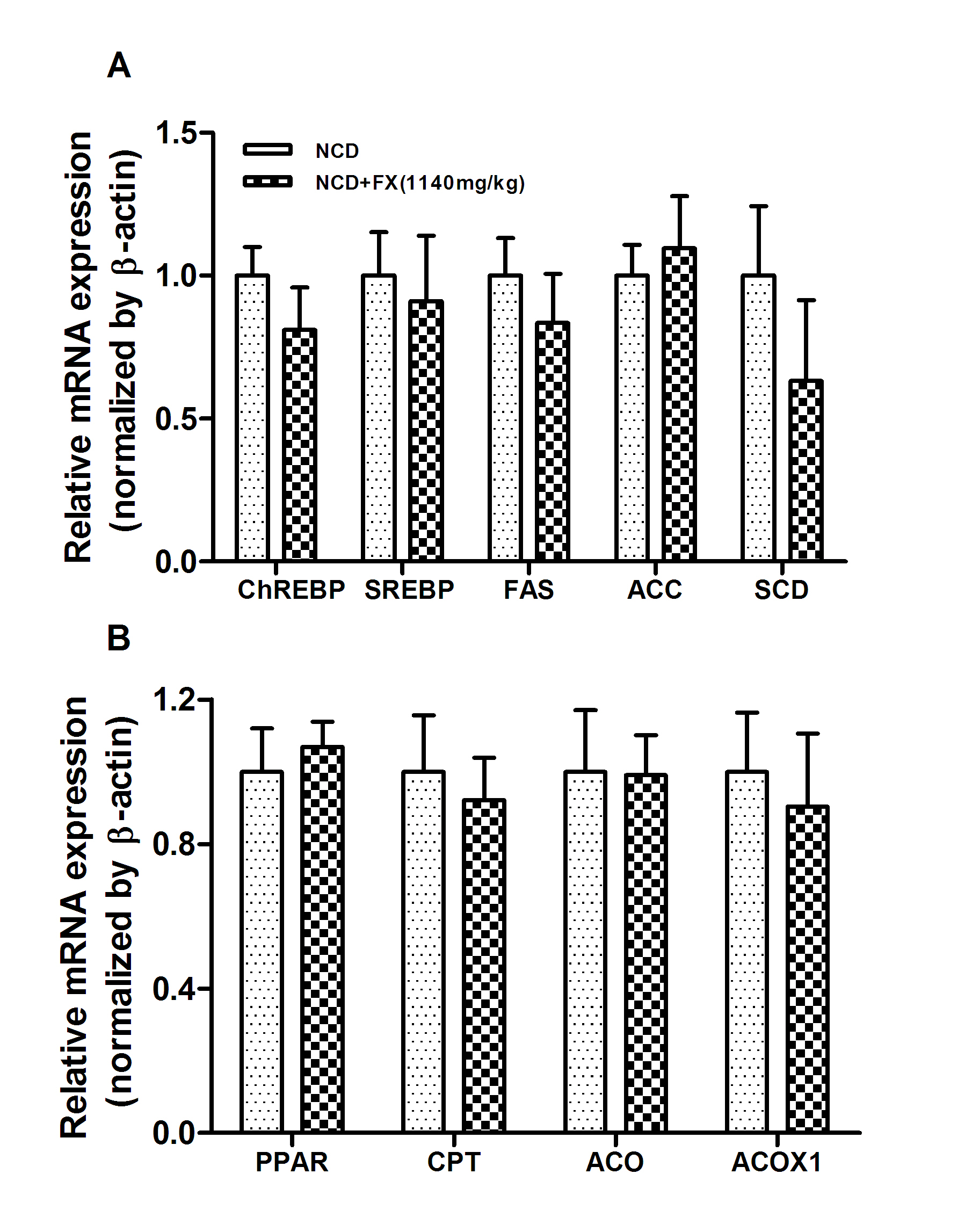

Supplement: Figure S2 — Relative expression of lipogenic, lipolytic genes of liver in NCD treated with FX (1140 mg/kg). Data given are mean ± SE. N = 5. (TIF) [file pone.0061499.s002.tif]

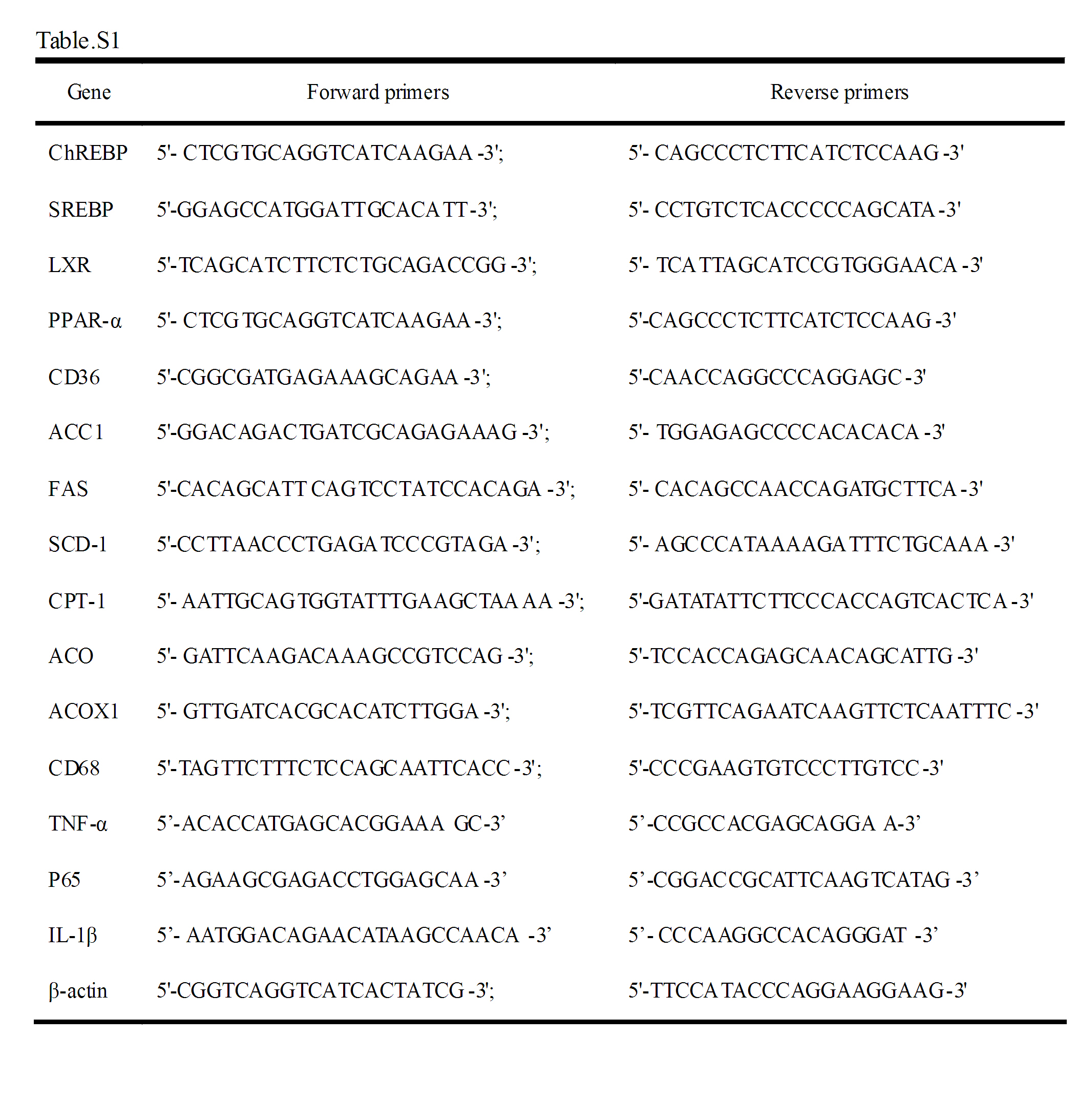

Supplement: Table S1 — The oligonucleotide primers used. (TIF) [file pone.0061499.s003.tif]

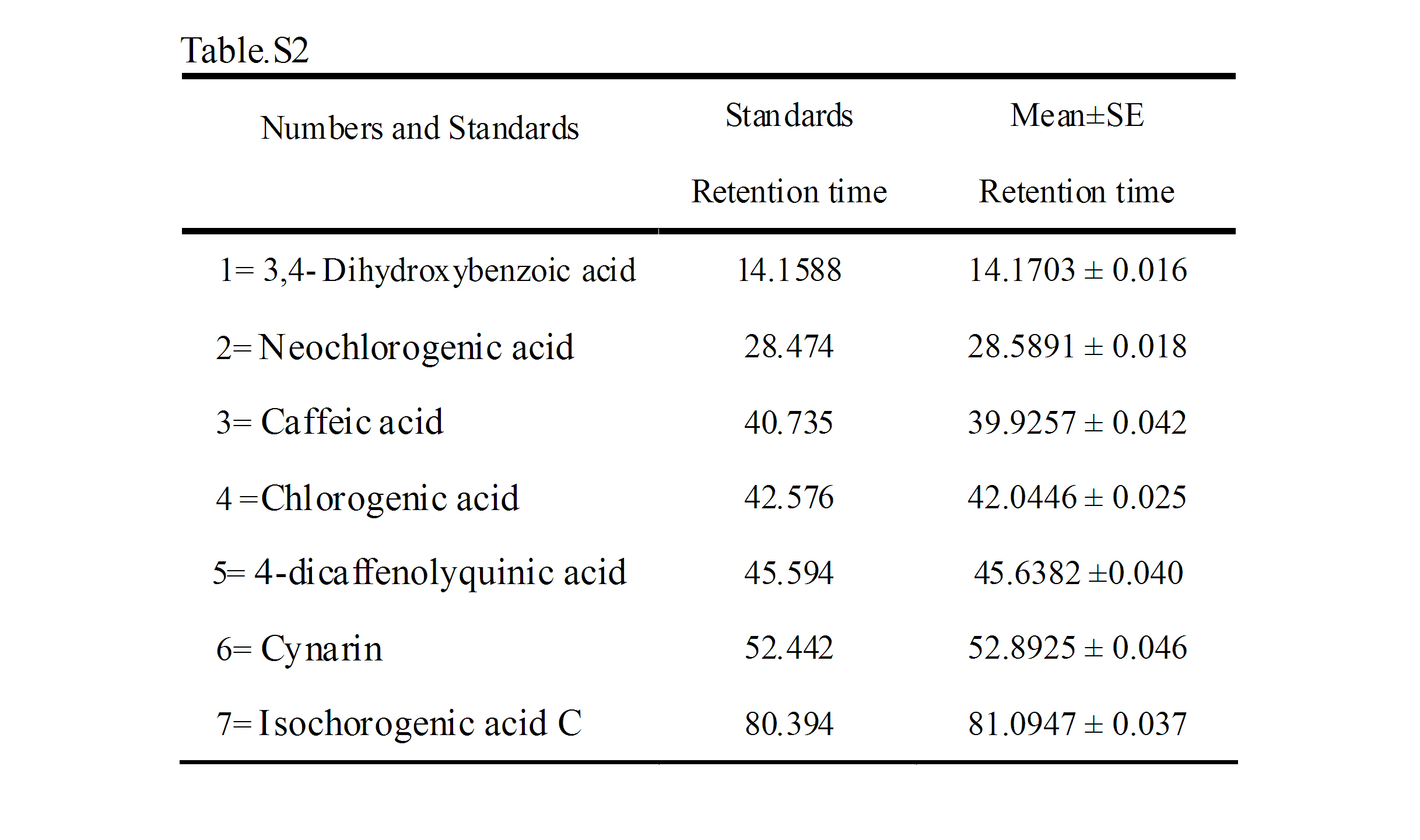

Supplement: Table S2 — The retention time of the seven marker compounds in the three batches of FX decoction was shown as Mean ± SE. (TIF) [file pone.0061499.s004.tif]
